# *SlWRKY80*-mediated jasmonic acid pathway positively regulates tomato resistance to saline–alkali stress by enhancing spermidine content and stabilizing Na^+^/K^+^ homeostasis

**DOI:** 10.1093/hr/uhae028

**Published:** 2024-01-24

**Authors:** Chunyu Shang, Xiaoyan Liu, Guo Chen, Hao Zheng, Abid Khan, Guobin Li, Xiaohui Hu

**Affiliations:** College of Horticulture, Northwest A&F University, Yangling, Shaanxi, 712100, China; Key Laboratory of Protected Horticultural Engineering in Northwest, Ministry of Agriculture and Rural Affairs, Yangling, Shaanxi, 712100, China; Shaanxi Protected Agriculture Engineering Technology Research Centre, Yangling, Shaanxi, 712100, China; College of Horticulture, Northwest A&F University, Yangling, Shaanxi, 712100, China; Key Laboratory of Protected Horticultural Engineering in Northwest, Ministry of Agriculture and Rural Affairs, Yangling, Shaanxi, 712100, China; Shaanxi Protected Agriculture Engineering Technology Research Centre, Yangling, Shaanxi, 712100, China; College of Horticulture, Northwest A&F University, Yangling, Shaanxi, 712100, China; Key Laboratory of Protected Horticultural Engineering in Northwest, Ministry of Agriculture and Rural Affairs, Yangling, Shaanxi, 712100, China; Shaanxi Protected Agriculture Engineering Technology Research Centre, Yangling, Shaanxi, 712100, China; College of Horticulture, Northwest A&F University, Yangling, Shaanxi, 712100, China; Key Laboratory of Protected Horticultural Engineering in Northwest, Ministry of Agriculture and Rural Affairs, Yangling, Shaanxi, 712100, China; Shaanxi Protected Agriculture Engineering Technology Research Centre, Yangling, Shaanxi, 712100, China; Department of Horticulture, The University of Haripur, Haripur, 22620, Pakistan; College of Horticulture, Northwest A&F University, Yangling, Shaanxi, 712100, China; Key Laboratory of Protected Horticultural Engineering in Northwest, Ministry of Agriculture and Rural Affairs, Yangling, Shaanxi, 712100, China; Shaanxi Protected Agriculture Engineering Technology Research Centre, Yangling, Shaanxi, 712100, China; College of Horticulture, Northwest A&F University, Yangling, Shaanxi, 712100, China; Key Laboratory of Protected Horticultural Engineering in Northwest, Ministry of Agriculture and Rural Affairs, Yangling, Shaanxi, 712100, China; Shaanxi Protected Agriculture Engineering Technology Research Centre, Yangling, Shaanxi, 712100, China

## Abstract

Saline–alkali is an important abiotic stressor influencing tomato production. Exogenous methyl jasmonate (MeJA) is well known to increase tomato resistance to a variety of stresses, although its exact mechanism is yet unknown. In this study we confirmed that 22.5 μmol/l MeJA could significantly improve the saline–alkali stress resistance of tomato. Saline–alkali (300 mM) stress increased the endogenous MeJA and jasmonic acid (JA) contents of tomato by 18.8 and 13.4%, respectively. Exogenous application of 22.5 μmol/l MeJA increased the endogenous MeJA and JA contents in tomato by 15.2 and 15.9%, respectively. Furthermore, we found an important transcription factor, *SlWRKY80*, which responded to MeJA, and constructed its overexpressing and knockout lines through genetic transformation. It was found that *SlWRKY80* actively regulated tomato resistance to saline–alkali stress, and the spraying of exogenous MeJA (22.5 μmol/l) reduced the sensitivity of *SlWRKY80* knockout lines to saline–alkali stress. The SlWRKY80 protein directly combines with the promoter of *SlSPDS2* and *SlNHX4* to positively regulate the transcription of *SlSPDS2* and *SlNHX4*, thereby promoting the synthesis of spermidine and Na^+^/K^+^ homeostasis, actively regulating saline–alkali stress. The augmentation of JA content led to a notable reduction of 70.6% in the expression of *SlJAZ1*, and the release of the SlWRKY80 protein interacting with SlJAZ1. In conclusion, we revealed the mechanism of exogenous MeJA in tomato stress resistance through multiple metabolic pathways, elucidated that exogenous MeJA further promotes spermidine synthesis and Na^+^/K^+^ homeostasis by activating the expression of *SlWRKY80*, which provides a new theoretical basis for the study of the JA stress resistance mechanism and the production of tomato.

## Introduction

Around the world, the tomato (*Solanum lycopersicum* L.) is the most widely grown and consumed horticultural crop. In addition to moderate salt sensitivity, tomatoes are also susceptible to abiotic stresses such as saline–alkali, which negatively affect their growth [41]. However, there is a problem of salinization on 33% of the world’s arable land, a condition that significantly impedes tomato productivity, rendering it a primary environmental concern thwarting global agricultural development of a high-quality nature [11]. Therefore, studying how tomato plants respond to saline–alkali stress is essential to improving tomato quality and yield.

Plants activate various mechanisms and trigger changes in endogenous phytohormones to response to saline–alkali stress, including jasmonic acid (JA) [74], abscisic acid [13, 39, 66, 74], brassinosteroids edna [32], and ethylene [35] etc. Plants under salt stress exhibit positive effects on JA, and endogenous JA content is enhanced and JA signaling is activated under salt stress [69]. The high JA-accumulating tomato mutant *res* exhibits stronger salt tolerance [16], while *def-1* (JA-deficient mutant) was salt-sensitive [2]. Similarly, studies on JA-related mutants in wheat [75], rice [17], and corn (maize) [3] have shown that JA is associated with salt stress responses. Methyl jasmonate (MeJA) exhibits a similar function to JA in participating in plant stress resistance. MeJA enters the plant through the stomata, is hydrolyzed into JA by esterases in the cytoplasm, and facilitates long-distance signal propagation and interplant communication. This process induces defense responses in nearby plants, fortifying their resilience as well [60]. For instance, exogenously applied MeJA enhances plants’ salt tolerance, either by maintaining reactive oxygen species (ROS) homeostasis, or by stabilizing the ion equilibrium [69]. The proteins of JAZ, which contain a jasmonate ZIM domain, act as repressors that participate in multiple signaling pathways and can bind to transcription factors or other corepressor proteins, linking the JA signaling pathway with other signaling pathways. COI1, an F-box protein, is a core component of the JA signaling receptor [5, 36].

Hormones exert direct effects on transcription factors, including the WRKY gene family, one of the first and largest transcriptional regulators to be identified. Furthermore, WRKY can mediate the influence of JAZ genes on *Arabidopsis thaliana*’s resistance to *Botrytis cinerea* [23]. Additionally, *CaWRKY40* in chili pepper suppresses the expression of the JA signaling repressor *JAZ8*, thereby enhancing disease resistance [48]. As well as being involved in stress response, the WRKY proteins bind to the W-box (TTGACT/C) sequence in the promoter regions of target genes [49]. Factors including *AtWRKY25/33* [25], *AtWRKY8* [21], *AtWRKY46* [14], *MdWRKY100* [40], and *AcWRKY28* [62] along with negative regulators such as *AtWRKY15* [55], *PalWRKY77* [26], *ZmWRKY20/115* [10], and *OsWRKY53* [70], are reported to be involved in salt stress responses. Notably, according to a recent study, WRKY transcription factors and their feedback loops function as central nodes in salt-responsive gene regulatory networks, indicating that WRKYs play an indispensable role in plant responses to salt stress [61].

There are 83 known *SlWRKY* genes in tomato [22]. A significant role is played by *SlWRKY80* in the plant’s disease resistance, answering the call of signals from salicylic acid (SA) as well as JA [44]. Group III of the SlWRKY family genes actively participate in the response to abiotic stress [7], and the group III subfamily of the tomato SlWRKY family encompasses a collective sum of eight genes, specifically identified as *SlWRKY30*, *SlWRKY41*, *SlWRKY52*, *SlWRKY53*, *SlWRKY54*, *SlWRKY59*, *SlWRKY80*, and *SlWRKY81* [12]. The interaction of *SlWRKY30* and *SlWRKY80* further bolsters the resistance of *SlPR-STH2* to bacterial wilt [12]. Yet the biological function of *SlWRKY80* under abiotic threats such as saline–alkali stress remains unelucidated, and the stress response mechanisms of *SlWRKY80* are not fully understood. Other genes in the WRKY family such as *SlWRKY33* [76], *SlWRKY39* [53], *SlWRKY8* [15], *SlWRKY79* [19], and *SlWRKY23* [52] are associated with salt stress, while *SlWRKY28* [57] has been associated with alkaline stress. However, current research on the *SlWRKY* genes in tomato under saline–alkali stress still needs further clarification. Shedding light on the pivotal function of WRKY genes under such adversities, and comprehending their operational mechanisms, will offer theoretical foundations for the enhancement of tomato resistance breeding, and has crucial significance.

Spermidine (Spd) is a free compound existing within plants, a type of polyamine, and has a prominent role in preventing ionic toxicity and reducing salt–alkali stress. Overexpression of *SlSPDS2*, which is involved in Spd synthesis, reduces Na^+^/K^+^ and H_2_O_2_ levels, mitigated ionic toxicity of tomato [56]. In response to abiotic stresses like cold, freezing, and salinity, Spd synthesis-related genes are overexpressed, along with transcription factors like WRKY that are increased [27]. Increasing the expression of *PtSPD* also significantly improved the tolerance of a member of the *Populus* genus, *P. davidiana*, to saline–alkali stress [57]. All these data indicate that the genes for *WRKT* TF and* SPDS* gene play important roles in plant resistance to saline–alkali stress, but the mechanism of action between *WRKT* TF and *SPDS* gene is still unclear.

Additionally, plants express genes involved in ion transport in the plasma membrane to combat saline–alkali stress, such as *SOS1*, *HKT1.1*, *HKT1.2*, *NHX1*, and *NHX4* [8, 45]. These functional genes expel excessive sodium ions from the cells or sequester them into vacuoles, reducing sodium ion accumulation within the cells. Concurrently, the expression of potassium channel protein-encoding genes *LKT1*, *HAK20*, and *NHX2* is induced, which in turn promotes potassium ion absorption and transport. By doing so, the saline–alkali stress-induced ionic toxicity is lessened and the Na^+^/K^+^ ratio is decreased [18]. NHXs are membrane-localized proteins that play roles in maintaining the Na^+^/K^+^ and pH homeostasis within cells. A reduction in the concentration of Na^+^ in the cytosol is achieved by removing or enclosing Na^+^ ions from the cytoplasm. By activating potassium ion channel proteins and increasing K^+^ content, the NHX proteins preserve Na^+^/K^+^ ion homeostasis, constituting an essential mechanism for mitigating ionic toxicity and enhancing saline–alkali stress resistance [8]. Furthermore, *AtWRKY75* is able to bind to the promoter of *AtSOS1* in *Arabidopsis*, thereby regulating the expression of *AtSOS1* [38].

We observed that the *SlWRKY* gene family of tomato showed significant responses to saline–alkali stress, and a certain concentration of exogenous MeJA could enhance tomato saline–alkali tolerance. Keeping in view the importance of the *SlWRKY* gene family, we selected the *SlWRKY80* gene through transcriptome analysis. *SlWRKY80*-overexpressing and knockout lines were obtained through genetic transformation, and functional validation was conducted under saline–alkali stress. This experimental study found that the transcription factor *SlWRKY80* was significantly upregulated under saline–alkali stress, and the promoter of *SlWRKY80* can respond to both saline–alkali and exogenous MeJA signals simultaneously. This study aimed to investigate how exogenous MeJA participates in tomato saline–alkali tolerance through the regulation of *SlWRKY80*, and in order to find a theoretical explanation for tomato tolerance to saline–alkali stress we studied the relationship between *SlWRKY80*, JA signal transduction, Spd synthesis, and the balance of Na^+^/K^+^ homeostasis.

## Results

### Exogenous methyl jasmonate has dual effects on tomato saline–alkali stress

**Fig. 1:**
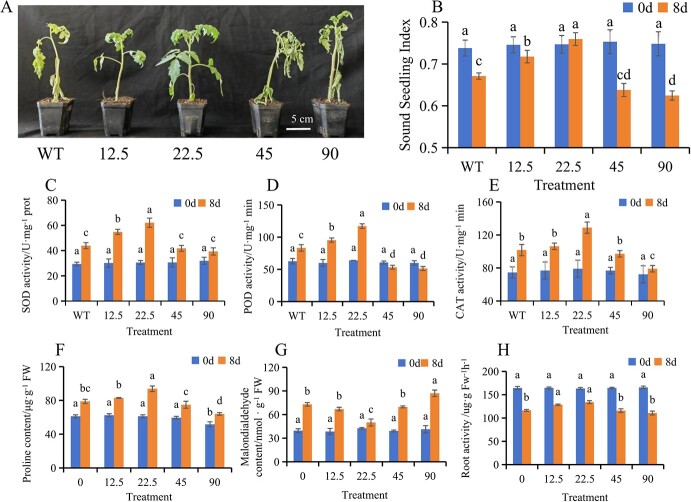
Impact of different concentrations of exogenous MeJA on tomato’s resilience to saline-alkali stress. **A** Phenotypes of tomato plants sprayed with different concentrations of exogenous MeJA under saline-alkali treatment. **B** Sound seedling index. **C**–**E** SOD, POD, and CAT enzyme activities. **F** Proline content. **G** Malondialdehyde content. **H** Root activity. According to the LSD test, significant differences are indicated by lowercase letters (*P* < 0.05), and values indicate the mean across three biological replicates.

Several concentrations of MeJA were applied to wild-type (WT) tomato seedlings to investigate their response to saline–alkali stress. With higher concentrations of exogenous MeJA under saline–alkali stress, the tolerance of tomato seedlings initially exhibited an increasing trend followed by a decrease (Fig. 1A). Further analysis showed that when the concentration of exogenous MeJA was 22.5 μmol/l, the morphological indicators of stem diameter and plant height (Supplementary Data Fig. S1) and physiological indicators such as SOD and POD (Fig. 1B–F, Supplementary Data Fig. S2) were significantly higher than in other treatment groups, while the change trend of malondialdehyde content was opposite (Fig. 1G). Therefore, exogenous MeJA has dual effects on tomato saline–alkali stress, while spraying exogenous 22.5 μmol/l MeJA can significantly affect tomato resistance to saline–alkali stress.

### *SlWRKY80* responded to saline–alkali stress and methyl jasmonate

**Fig. 2:**
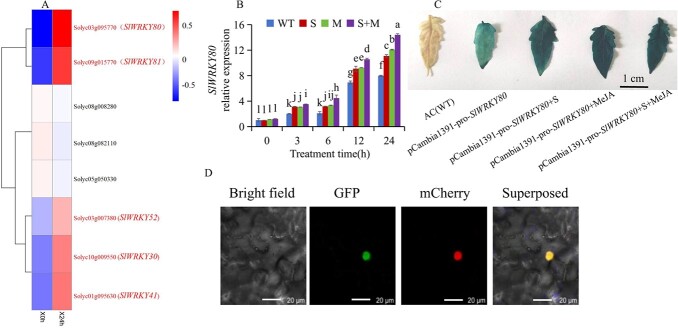
Gene expression analysis of the third subgroup of SlWRKYs in tomato under saline–alkali stress, response of *SlWRKY80* to saline–alkali stress and MeJA, and the subcellular localization of *SlWRKY80*. **A** Heat map of gene expression in the third subgroup of tomato SlWRKYs under saline–alkali stress. **B***SlWRKY80* expression under S, M and S + M conditions. S represents 300 mM saline-alkali stress; M represents exogenous spraying of 22.5 μmol/l MeJA; S + M represents saline-alkali stress and exogenous spraying of 22.5 μmol/l MeJA. According to the LSD test, significant differences are indicated by lowercase letters (*P* < 0.05), and values indicate the mean across three biological replicates. **C** GUS staining map of pCAMBIA1391-pro-*SlWRKY80* transgenic material under saline–alkali, exogenous MeJA spraying, and simultaneous treatment with saline–alkali and exogenous MeJA. S, saline–alkali; MeJA concentration was 22.5 μmol/l. **D** Subcellular localization of SlWRKY80 protein.

In tomato, *SlWRKY80* significantly responded to saline–alkali stress when eight genes in the third subfamily of the SlWRKY family were examined (Fig. 2A) [12]. We further examined the relative expression level of *SlWRKY80* in tomato seedlings treated with saline–alkali at 3, 6, 12, and 24 h. In the presence of saline–alkali treatment (S), *SlWRKY80* expression levels were significantly increased. When treated with saline–alkali and exogenous MeJA (S + M), a significant increase in *SlWRKY80* expression was observed (Fig. 2B).

To verify whether the *SlWRKY80* promoter is regulated by saline–alkali and MeJA, we subjected pCAMBIA1391-pro-*SlWRKY80*-positive lines to 300 mM saline–alkali treatment (+S), exogenous spraying of 22.5 μmol/l MeJA (+MeJA), and simultaneous treatment with 300 mM saline–alkali and exogenous spraying of 22.5 μmol/l MeJA (+S + MeJA). We found that the depth of GUS staining gradually deepened (Fig. 2C). Grayscale analysis of GUS-stained images yielded the same results (Supplementary Data Fig. S3). The data also showed that S + M treatment made the GUS staining of pCAMBIA1391-pro-*SlWRKY80* transgenic material significantly higher than that of other treatments (Supplementary Data Fig. S3), indicating that the promoter of *SlWRKY80* responded to both saline–alkali treatment and exogenous MeJA, and S + MeJA treatment could make the promoter of *SlWRKY80* respond more significantly. In the analysis of the 2000-bp promoter upstream of *SlWRKY80*, we also found that there were four *cis*-acting elements responding to MeJA (Supplementary Data Fig. S4), which was also consistent with the GUS staining results in this experiment (Fig. 2C). SlWRKY80 protein and GFP were fused under the PBI121 vector in order to determine its subcellular localization. A fluorescence microscope analysis revealed that GFP fluorescence was only found in the nucleus (Fig. 2D), indicating that SlWRKY80 protein belongs to the nucleus.

### *SlWRKY80* positively regulates saline–alkali stress

*SlWRKY80* was further tested under saline–alkali stress. We obtained *SlWRKY80*-overexpressing lines 80OE-1 and 80OE-3 (Supplementary Data Fig. S5) and knockout lines 80CR-3 and 80CR-4 (Supplementary Data Fig. S6) by genetic transformation.

Before saline–alkali stress, the lines did not differ significantly in plant height (Supplementary Data Fig. S7A), soluble sugar (Supplementary Data Fig. S8A), and soluble protein content (Supplementary Data Fig. S8B). However, stem diameter (Supplementary Data Fig. S7B) and leaf area (Supplementary Data Fig. S7C) of the *SlWRKY80*-overexpressing lines showed no significant differences. Carotenoids (Supplementary Data Fig. S8C), chlorophyll a (Supplementary Data Fig. S8D), and total chlorophyll content (Supplementary Data Fig. S8F) were significantly higher than those of WT, while the *SlWRKY80*-knockout line showed the opposite trend.

**Fig. 3:**
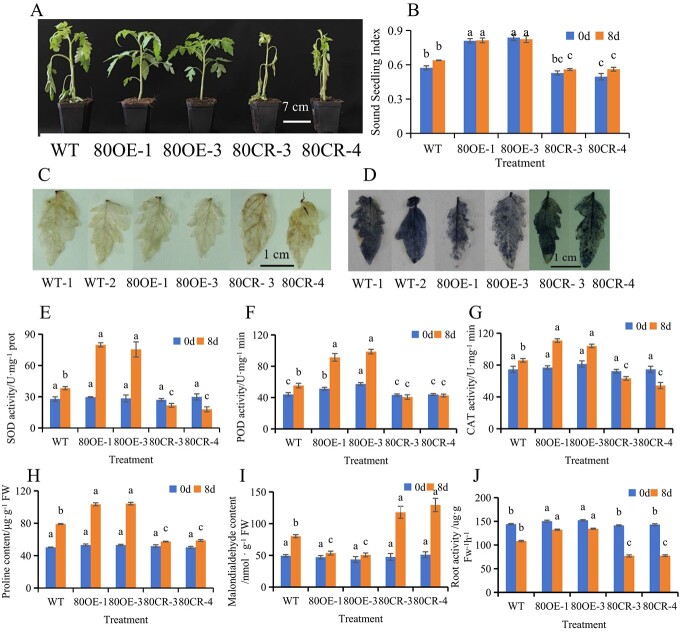
Morphology and physiological responses of WT and *SlWRKY80* transgenic plants to saline–alkali stress. **A** Phenotypic image of *SlWRKY80* transgenic tomato lines treated with saline–alkali stress. Take photos of tomato seedlings after 8 days of saline-alkali treatment. **B** Sound seedling index. **C**, **D** DAB and NBT holding charts on the eighth day of saline-alkali stress. **E**–**G** SOD, POD, and CAT activity. **H**–**J** Proline content, malondialdehyde content, and root activity. According to the LSD test, significant differences are indicated by lowercase letters (*P* < 0.05), and values indicate the mean across three biological replicates.

The *SlWRKY80* transgenic and WT tomato seedlings were treated with 300 mM saline–alkali solution, and obvious phenotypes were observed on the eighth day. Compared with WT, *SlWRKY80*-overexpressing lines showed significantly better growth, and the growth of *SlWRKY80* knockout lines was the worst (Fig. 3A). The sound seedling index (Fig. 3B), DAB staining (Fig. 3C), and NBT staining (Fig. 3D) also showed the same results. In contrast to the WT, except for the lower content of malondialdehyde in the *SlWRKY80*-overexpressing line, its morphological and physiological characteristics were significantly higher, while the *SlWRKY80*-knockout line showed the opposite results, indicating that overexpression of *SlWRKY80* significantly improved stem diameter, leaf area, antioxidant capacity, root activity, etc. of the tomato plants. Saline–alkali stress stimulated *SlWRKY80* expression (Fig. 3E–J). Therefore, we concluded that *SlWRKY80* can actively regulate saline–alkali stress.

### Exogenous application of methyl jasmonate reduces the sensitivity of *SlWRKY80* knockout lines to saline–alkali stress

There is a sensitivity to saline–alkali stress in *SlWRKY80* knockout lines, and exogenous 22.5 μmol/l MeJA significantly enhances the tomato’s resistance to saline–alkali stress. We asked whether exogenous 22.5 μmol/l MeJA reduces the *SlWRKY80*-knockout lines’ sensitivity to saline–alkali stress.

**Fig. 4:**
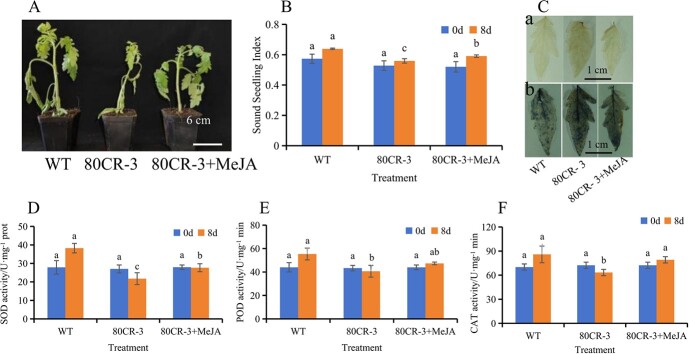
Exogenous application of MeJA reduces the sensitivity of *SlWRKY80* knockout lines to saline-alkali stress. **A** Phenotypic map of the effect of exogenous MeJA on the *SlWRKY80* knockout lines on the eighth day after treatment. **B** Sound seedling index of WT, 80CR-3, and 80CR-3+MeJA. **C** DAB (a) and NBT (b) staining of WT, 80CR-3, 80CR-3+MeJA on the eighth day of saline–alkali treatment. **(D**–**F)** SOD, POD, and CAT activities of WT, 80CR-3, 80CR-3 + Break. According to the LSD test, significant differences are indicated by lowercase letters (*P* < 0.05), and values indicate the mean across three biological replicates.

Following 8 days of saline–alkali treatment, exogenous 22.5 μmol/l MeJA significantly reduced the sensitivity of *SlWRKY80* knockout lines to saline–alkali stress. Saline–alkali stress affected 80CR-3 lines the most compared with WT, and the damage of the 80CR-3 lines was alleviated by saline–alkali stress after spraying exogenous 22.5 μmol/l MeJA (80CR-3 + MeJA) (Fig. 4A). Before treatment, SOD activity (Fig. 4D), POD activity (Fig. 4E), and CAT activity (Fig. 4F) did not differ significantly. Sound seedling index and SOD, POD, and CAT activities of tomato seedlings in the 80CR-3 + MeJA group were significantly higher than in the 80CR-3 group on the eighth day after treatment, while DAB staining (Fig. 4Ca) and NBT staining (Fig. 4Cb) showed the same results. These results showed that exogenous 22.5 μmol/l MeJA could significantly reduce the sensitivity of *SlWRKY80* knockout lines to saline–alkali stress.

### SlWRKY80 participates in the stress resistance pathway of jasmonic acid to saline–alkali stress through its interaction with SlJAZ1

**Fig. 5:**
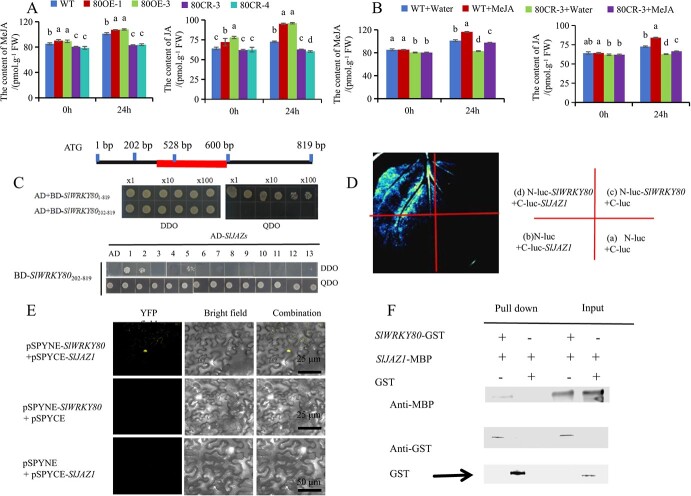
Overexpression of *SlWRKY80* increases endogenous MeJA content and interacts with transcriptional suppressor SlJAZ1. **A** Endogenous MeJA content of WT and SlWRKY80 transgenic lines pre- and 24 h-post-processing. **B** Content of endogenous MeJA in WT, WT + MeJA, 80CR-3, and 80CR-3 + MeJA pre- and 24 h-post-processing. According to the LSD test, significant differences are indicated by lowercase letters (*P* < 0.05). **C** Self-activation verification of SlWRKY80 and verification of the interaction relationship between SlWRKY80 and tomato SlJAZ family through the Y2H test *in vitro*. **D**, **E** Interaction between SlWRKY80 and SlJAZ1 through the BiFC test and LCI *in vivo*. **F** Verification of the interaction relationship between SlWRKY80 and SlJAZ1 through a pull-down test *in vitro*.

A study was conducted to determine if endogenous JA and MeJA are impacted by saline–alkali stress in *SlWRKY80* transgenic lines. We found that *SlWRKY80*-overexpressing lines had significantly increased contents of endogenous MeJA and JA in tomato. After 24 h of saline–alkali treatment, *SlWRKY80*-overexpressing lines contained significantly more MeJA and JA than WT, while the *SlWRKY80* knockout lines were the opposite (Fig. 5A). Therefore, *SlWRKY80*-overexpressing lines had significantly increased endogenous MeJA and JA levels, while the knockout lines showed the opposite effect. MeJA and JA levels were lowest in the 80CR-3 group, followed by 80CR-3 + MeJA, and highest in the WT group of tomatoes treated with exogenous 22.5 μmol/l MeJA and saline–alkali (Fig. 5B). The 300 mM saline–alkali stress increased the endogenous MeJA and JA contents in tomato by 18.8 and 13.4%, respectively. Exogenous application of 22.5 μmol/l MeJA increased the endogenous MeJA and JA contents in tomato by 15.2 and 15.9%, respectively. In addition, the findings also revealed a significant increase in the expression of JA synthesis-related genes, specifically *SlLoxD* and *SlAOC*, upon exogenous MeJA spraying (Supplementary Data Fig. S11). Consequently, it can be inferred that exogenous application of 22.5 μmol/l MeJA could significantly increase the content of endogenous JA.

JA content is negatively correlated with the expression of SlJAZs [30, 50]. Therefore, we have focused on the transcriptional suppressor genes SlJAZs in tomato, which are also significant in signal transduction. We verified the protein level interaction between SlWRKY80 and SlJAZ1, SlJAZ2 and SlJAZ5 through yeast two-hybrid (Y2H) assays (Fig. 5C). We verified it by luciferase complementary imaging (LCI) (Fig. 5D), bimolecular fluorescence complementation (BiFC) (Fig. 5E), and pull-down (Fig. 5F) assays *in vivo* and *in vitro*, and found that only SlJAZ1 could get positive results under verification by these molecular means. Therefore, we conclude that SlWRKY80 is involved in the resistance pathway of JA to saline–alkali stress through the interaction with SlJAZ1.

### SlWRKY80 binds the promoter of *SlSPDS2* to promote spermidine synthesis

**Fig. 6:**
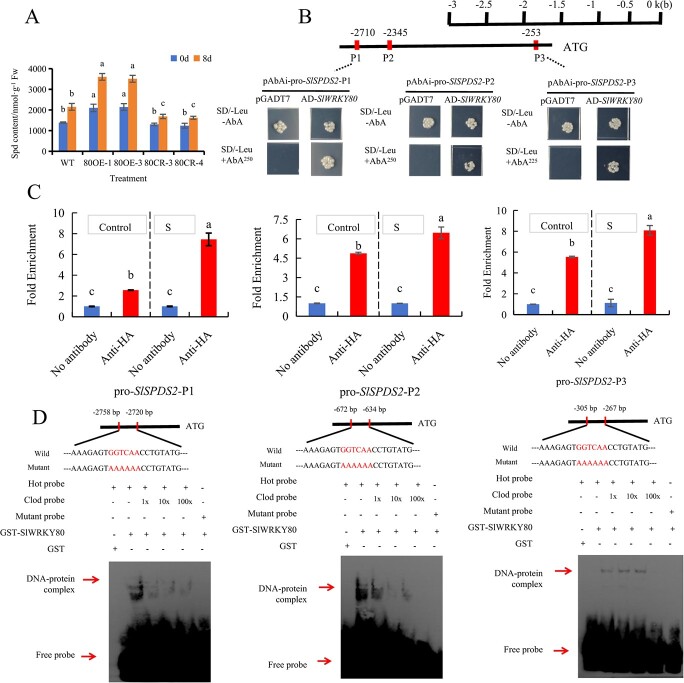
Binding of *SlWRKY80* to the promoter region of *SlSPDS2* and promotion of Spd synthesis. **A** Spd content in *SlWRKY80* transgenic tomato materials before and after saline–alkali treatment. **B** Y1H assay. **C** SlWRKY80 interaction with the *SlSPDS2* promoter confirmed by ChIP–qPCR assay. The three W-boxes within the 3000-bp upstream promoter of *SlSPDS2* are denoted as pro-*SlSPDS2*-P1, pro-*SlSPDS2*-P2, and pro-*SlSPDS2*-P3. According to the LSD test, significant differences are indicated by lowercase letters (*P* < 0.05), and values indicate the mean across three biological replicates. **D** SlWRKY80 interacts with the *SlSPDS2* promoter *in vitro*, as shown by EMSA.

To further investigate how saline–alkaline stress regulates *SlWRKY80*, we measured the content of endogenous Spd of tomato seedlings of different lines pre- and post-saline–alkali. There was significantly reduced endogenous Spd content in *SlWRKY80* knockout lines on the eighth day. At the same time, overexpression of *SlWRKY80* before saline–alkali treatment could significantly increase the content of tomato Spd, which was further enhanced after 8 days of saline–alkali treatment (Fig. 6A). In addition, the expression trend of *SlSPDS2* was similar to that of endogenous Spd content (Supplementary Data Fig. S9).

To explore whether this enhancement is regulated by *SlWRKY80*, we analyzed the 3000-bp promoter upstream of *SlSPDS2*, a functional gene for Spd synthesis, and found that it has three W-boxes. Subsequently, we confirmed that SlWRKY80 could directly bind to the three W-boxes in the *SlSPDS2* promoter by *in vivo* and *in vitro* verification methods such as the yeast one hybrid (Y1H) assay (Fig. 6B), chromatin immunoprecipitation (ChIP)–qPCR (Fig. 6C), and the electrophoretic mobility shift assay (EMSA) (Fig. 6D). To sum up, tomato can enhance resistance to saline–alkali stress by enhancing the expression of *SlWRKY80* and regulating the synthesis of Spd.

### SlWRKY80 binds to the *SlNHX4* promoter and reduces Na^+^/K^+^ ratio

**Fig. 7:**
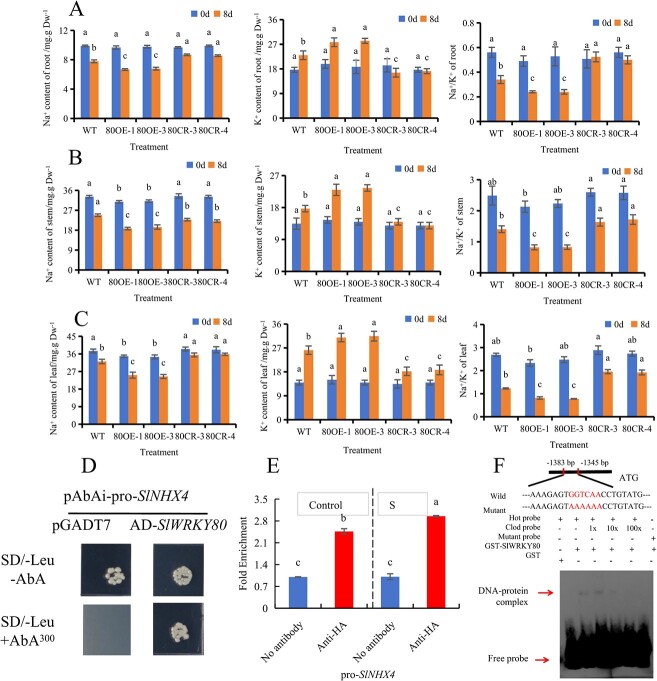
By binding to the promoter of *SlNHX4*, *SlWRKY80*-overexpressing lines decrease the Na^+^/K^+^ ratio in tomato. **A**–**C** Na^+^ and K^+^ contents and Na^+^/K^+^ ratio of root, stem, and leaf. According to the LSD test, significant differences are indicated by lowercase letters (*P* < 0.05), and values indicate the mean across three biological replicates. **D** Y1H assay. **E** ChIP–qPCR. **F** SlWRKY80 interacts with the *SlNHX4* promoter *in vitro*, as shown by EMSA.

In order to further investigate whether *SlWRKY80* regulates saline–alkali stress by regulating Na^+^ and K^+^ transport, we found through experiments that after 8 days of saline–alkali stress treatment the Na^+^ content, K^+^ content, and Na^+^/K^+^ in the root (Fig. 7A), stem (Fig. 7B), and leaf (Fig. 7C) of WT and *SlWRKY80* transgenic lines showed the same trend. *SlWRKY80-*overexpressing lines had a significantly reduced Na^+^/K^+^ ratio, promoting K^+^ absorption and Na^+^ outflow. Through direct comparison of the evolutionary trees of tomato *SlNHX* and *Arabidopsis AtNHX*, it was found that the genetic relationship between *AtNHX1*, *AtNHX2*, and *SlNHX4* was as high as 82%, with the closest genetic relationship (Supplementary Data Fig. S4C), and the expression level of *SlNHX4* significantly increased in response to salt stress [73]. Reports have shown that *AtNHX1* and *AtNHX2* are located in vacuolar membranes and are responsible for regulating the transport of Na^+^ and K^+^ [8]. At the transcriptional level, we measured the relative expression of *SlNHX4*, which regulates ion transport. The *SlWRKY80*-overexpressing lines showed significantly increased relative expression of *SlNHX4*, while the *SlWRKY80* knockout lines showed the opposite effect. At the same time, the *SlWRKY80*-overexpressing lines further enhanced the promotion of *SlNHX4* expression after saline–alkali stress (Supplementary Data Fig. S10).

To further explore the mechanism of *SlWRKY80* regulating Na^+^ and K^+^ homeostasis, we conducted a variety of studies on the functional genes regulating Na^+^ and K^+^ transport, and finally found that *SlNHX4* may be directly regulated by SlWRKY80. By analyzing the 3000-bp sequence upstream of the *SlNHX4* promoter, we found a W-box. Then we confirmed that SlWRKY80 can directly bind to the W-box in the *SlNHX4* promoter by yeast single hybridization (Y1H) (Fig. 7D), ChIP–qPCR (Fig. 7E), and EMSA (Fig. 7F) *in vivo* and *in vitro*. To sum up, we concluded that under saline–alkali stress the expression of *SlWRKY80* was enhanced, and then regulated the osmotic stress of tomato by regulating *SlNHX4* to further promote the absorption of K^+^ and the efflux of Na^+^ to reduce Na^+^/K^+^.

### Interaction between SlWRKY80 and SlJAZ1 inhibited the regulation of *SlSPDS2* and *SlNHX4 by SlWRKY80*

**Fig. 8:**
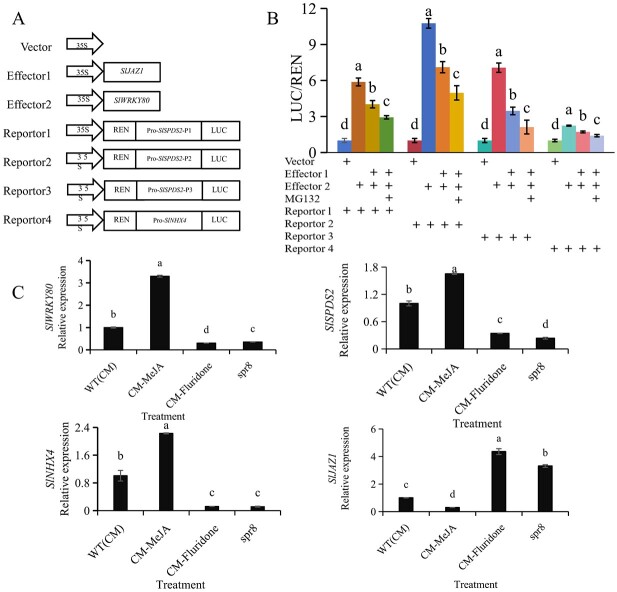
Results of the dual luciferase reporter assay. **A** A control plasmid, 35S:REN, was co-infiltrated as an interfering plasmid into *N. benthamiana* leaves. **B** Interaction between SlWRKY80 and SlJAZ1, resulting in the inhibition of *SlWRKY80* regulation of *SlSPDS2* (-P1/P2/P3) and *SlNHX4*, respectively. LUC/REN ratios were used to determine the ability of SlWRKY80 and SlJAZ1 to activate the reporter LUC gene. **C** Relative expression levels of *SlWRKY80*, *SlSPDS2*, *SlNHX4*, and *SlJAZ1* after exogenous spraying of MeJA or fluridone; *spr8* mutants were measured against the background of WT tomato CM. According to the LSD test, significant differences are indicated by lowercase letters (*P* < 0.05), and values indicate the mean across three biological replicates.

To further verify the effect of interaction between SlWRKY80 and SlJAZ1 on the regulation of downstream functional genes, we proved that SlWRKY80 positively regulated *SlSPDS2* and *SlNHX4* through the dual luciferase test, which was also consistent with the result of ChIP–qPCR (Fig. 6C and Fig. 7E), but when SlJAZ1 was present the regulation by SlWRKY80 of downstream *SlSPDS2* and *SlNHX4* decreased significantly (Fig. 8A and B). The interaction of SlJAZ1 and SlWRKY80 inhibited the regulation by SlWRKY80 of downstream *SlSPDS2* and *SlNHX4*. In addition, the JA synthesis mutant *spr8* is a mutant material based on the CM WT tomato, so in order to further validate this result at the transcriptional level, we treated CM WT tomato as follows: spraying 22.5 μmol/l MeJA or spraying 22.5 μmol/l fluridone, a JA synthesis inhibitor, was used to JA synthesize mutant *spr8*. The use of JA synthesis mutant *spr8* can infer whether *SlWRKY80* affects the entire pathway of JA synthesis or a certain segment of JA synthesis with *spr8* as the node, so it has certain reference significance. The relative expression levels of *SlWRKY80*, *SlSPDS2*, and *SlNHX4* in the fluridone group and the *spr8* group were significantly lower than those in the WT group, while the relative expression trend of *SlJAZ1* was opposite.(Fig. 8C). Exogenous MeJA promoted the expression of *SlWRKY80*, *SlSPDS2*, and *SlNHX4*, while inhibiting *SlJAZ1* led to a significant 70.6% decrease in *SlJAZ1* expression. This also confirms the negative regulatory relationship between *SlJAZ1* and *SlWRKY80* at the transcriptional level.

**Fig. 9:**
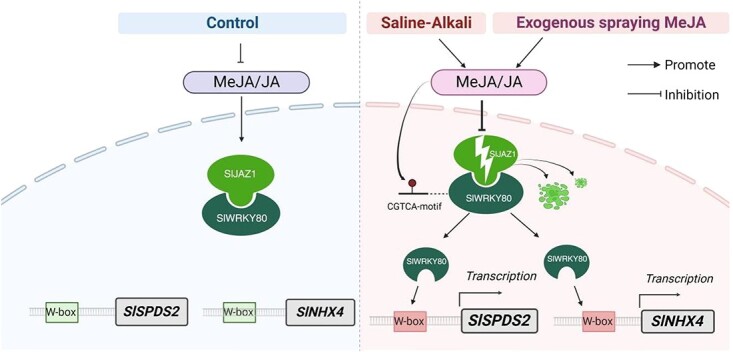
A working model for the saline–alkali reaction mediated by *SlWRKY80* through the JA pathway and the regulation of *SlSPDS2* and *SlNHX4* in tomato. The CGTCA motif is a MeJA-binding element.

To sum up, we can propose a model in which, under normal conditions, SlWRKY80 protein interacts with SlJAZ1 protein, and SlWRKY80 is bound by SlJAZ1. Under 300 mM saline–alkali stress, exogenous spraying of 22.5 μmol/l MeJA can significantly increase the content of endogenous MeJA and JA and accelerate the decomposition of SlJAZ1, which weakens or relieves the inhibitory effect of SlJAZ1 on SlWRKY80, thus releasing a large number of SlWRKY80 proteins to bind to the promoters of *SlSPDS2* and *SlNHX4* and activate the expression of these two downstream factors, hence promoting the synthesis of Spd and the homeostasis of Na^+^ and K^+^, thus regulating saline–alkali stress (Fig. 9).

## Discussion

### Saline–alkali stress resistance in tomato can be improved by exogenous methyl jasmonate at certain concentrations

JA plays a vital role, such as mechanical damage, disease, insect damage, drought, salt stress, high and low temperature [42, 71]. *Arabidopsis thaliana* [46], maize (*Zea mays* L.) [71] and *Dioscorea zingiberensis* [51] showed significantly increased internal JA content to enhance salt resistance. By exogenously applying JA to soybean (*Glycine max*), salt stress could be alleviated [54]. In addition, using MeJA exogenously can increase salt tolerance of plants by maintaining ROS or ion homeostasis [69].

In this study, exogenous MeJA increased resistance to saline–alkali-treated tomato at low concentration, and decreased resistance at high concentration. A 300 mM solution of saline–alkali was applied to tomato plants of the same growth potential, and different concentrations of MeJA were sprayed externally. When the concentration of MeJA was 22.5 μmol/l, the plant showed significantly higher morphological and physiological indicators (Fig. 1 and Supplementary Data Fig. S1). A dose of exogenous MeJA of 22.5 μmol/l significantly improves tomato protection against saline–alkali stress. When concentrations of exogenous MeJA are low, as a signaling molecule MeJA activates transcription factors in plants under stress [31]. In contrast, when exogenous MeJA is applied at high levels the plant absorbs excess MeJA, increasing its osmotic potential and causing osmotic stress. In addition, excessive exogenous MeJA can affect the balance between endogenous hormones, leading to abnormal regulation of plant hormones.

### *SlWRKY80* senses methyl jasmonate and saline–alkali signals and actively regulates saline–alkali stress

An important role of WRKY genes is to regulate the response of plants to biotic and abiotic stresses. The group III subfamily of tomato SlWRKY mainly responds to abiotic stresses in tomato [12, 24]. Toward a deeper understanding of how MeJA protects tomato against saline–alkali stress, we found *SlWRKY80*, located in group III of SlWRKY through transcriptome analysis before and after saline–alkali stress (Fig. 2A). *SlWRKY80* expression was highest in the S + M group, followed by the S group, and both were significantly higher than in the WT group (Fig. 2B). Meanwhile, the GUS staining results of pCAMBIA1391-pro-*SlWRKY80* transgenic material also showed that the promoter of *SlWRKY80* responded to MeJA and saline–alkali stress (Fig. 2C). Similarly, SA induced the mulberry *MiWRKY53* promoter to activate the resistance of *MiWRKY53* to *Pseudomonas syringae* [43].

To better explore the mechanism of *SlWRKY80* under saline–alkali stress, we obtained *SlWRKY80*-overexpressing lines (Supplementary Data Fig. S5) and knockout lines (Supplementary Data Fig. S6) by transgenic methods. After 300 mM saline–alkali treatment, the growth of *SlWRKY80*-overexpressing lines was the best, and the growth of *SlWRKY80* knockout lines was the worst (Fig. 3A). DNB staining (Fig. 3C) and NBT staining (Fig. 3D) also showed that the staining of *SlWRKY80*-overexpressing lines was the lightest, and the staining of *SlWRKY80* knockout lines was the deepest, indicating that the *SlWRKY80*-overexpressing lines removed more ROS, which was also consistent with the results of SOD activity, POD activity, CAT activity (Fig. 3G), and other indicators on the eighth day of treatment; the other morphological and physiological indexes were the same. Above all, *SlWRKY80* can actively regulate saline–alkali stress and the *SlWRKY80* promoter was responsive to exogenous MeJA (22.5 μmol/l), which stimulated the expression of *SlWRKY80* and further regulated saline–alkali stress.

Another interesting phenomenon is that exogenous MeJA (22.5 μmol/l) spraying of *SlWRKY80* knockout lines (80CR-3) can significantly increase the seedling strength index, SOD activity, POD activity, and CAT activity (Fig. 4), indicating that exogenous MeJA (22.5 μmol/l) spraying can significantly reduce the sensitivity of *SlWRKY80* knockout lines to saline–alkali stress. This phenomenon may be due to the activation of multiple metabolic pathways by exogenous MeJA as a signal molecule; *SlWRKY80* is only an important transcription factor in exogenous MeJA resistance to saline–alkali stress. Similarly, The CmGST family of genes, including *CaGSTU3*, *CaGSTU7*, and others is believed to contribute to pumpkins’ cold resistance [1]. Therefore, a new reference for studying JA stress resistance mechanisms is provided by this study.

### *SlWRKY80* directly combines with *SlSPDS2* and *SlNHX4* promoters to regulate spermidine synthesis and Na^+^/K^+^ homeostasis

Spd is a polyamine compound that helps plants adapt to abiotic stress and grow and develop [34, 64]. Exogenous spraying of Spd protects membrane lipids from peroxidation, regulates polyamine metabolism, and strengthens the antioxidant system in tomato [20, 33, 67]. Elevated endogenous Spd levels in tomato plants have been shown to significantly enhance their tolerance to saline–alkali stress [56]. In addition, saline–alkali stress primarily disrupts cellular ion homeostasis, emphasizing the need to understand the mechanisms of Na^+^ absorption and transport in plants and identify candidate genes that promote ion homeostasis to enhance crop salt tolerance [9]. Therefore, we wondered whether *SlWRKY80* actively regulated saline–alkali stress was related to Spd synthesis and Na^+^/K^+^ homeostasis or not.

We discovered that *SlWRKY80*-overexpressing lines had significantly increased the Spd content, which was further enhanced after 8 days of saline–alkali treatment (Fig. 5A). Similar results were found in different tissue parts. The contents of K^+^ in root (Fig. 6A), stem (Fig. 6B), leaf (Fig. 6C) and other tissue parts of *SlWRKY80*-overexpressing lines were significantly higher than those of WT. Likewise, the opposite was true for *SlWRKY80* knockout lines. We found the Spd synthesis gene (*SlSPDS2*) and the *SlNHX4* gene related to Na^+^/K^+^ transport through the transcriptome and from the literature, with confirmation through *in vivo* and *in vitro* experiments (Y1H, EMSA, ChIP–qPCR) that SlWRKY80 binds to *SlSPDS2* (Fig. 5B–D) and *SlNHX4* promoters (Fig. 6D–F), respectively. In our previous research, we found that overexpression of *SlSPDS2* [56] and *SlSPMS* [63] can increase the endogenous free polyamine content in tomato seeds. This leads to the regulation of ion balance, the antioxidant enzyme system, and osmotic regulators under saline–alkali stress, thus enhancing the resistance of tomato seeds to saline–alkali stress.

Following saline–alkali treatment, the Na^+^/K^+^ ratio in the *SlWRKY80*- overexpressing lines was notably lower compared with the WT, whereas the opposite was observed in the *SlWRKY80* knockout lines (Fig. 7A–C). Additionally, *SlWRKY80* exhibited active regulation of saline–alkali stress (Fig. 4). Consequently, the *SlWRKY80*-overexpressing lines may actively respond to saline–alkali stress through its involvement in the transportation of Na^+^ and K^+^. In addition, the genetic relationship between *AtNHX1*, *AtNHX2*, and *SlNHX4* was as high as 82% (Supplementary Data Fig. S4C), the genetic relationship between *AtNHX1*, *AtNHX2*, and *SlNHX4* is very close, so *SlNHX4* may have similar physiological functions as *AtNHX1* and *AtNHX2*, and the expression level of *SlNHX4* significantly increased in response to salt stress [73]. *AtNHX1* and *AtNHX2* are located in vacuolar membranes and are responsible for regulating the transport of Na^+^ and K^+^ [8]. The expression level of *SlNHX4* was significantly upregulated in the saline–alkali treatment and in *SlWRKY80*-overexpressing lines in this experiment. Finally, this experiment validated the upstream relationship between *SlWRKY80* and *SlNHX4* through molecular experiments such as Y1H (Fig. 7D) and EMSA (Fig. 7F). Under salt stress, *NHX* promoted the entry of Na^+^ into the vacuole and the absorption of K^+^ in tomato [4, 8]. Therefore, an increase in the endogenous Spd content and decrease in the Na^+^/K^+^ ratio are helpful to improve the saline–alkali resistance of tomato. Thus, we conclude that *SlWRKY80* promotes the synthesis of Spd and Na^+^/K^+^ homeostasis by interacting with downstream *SlSPDS2* and *SlNHX4* to actively regulate saline–alkali stress.

### SlJAZ1 is inhibited in saline–alkali stress and releases more SlWRKY80 when interacting with it

Studies have shown that MeJA and JA influence plant growth, development, and stress responses. Increased endogenous JA can reduce salt damage in wheat [47], rapeseed [28], rice [6], and other crops by activating genes involved in the JA signaling pathway. Salt damage can be alleviated by JA in wheat [47] and rapeseed [28]. Endogenous JA content increased and its signal transduction was activated when salt damage occurred [69]. In our study, after 24 h of saline–alkali stress, the endogenous MeJA and JA contents of tomato were significantly increased. *SlWRKY80*-overexpressing lines had significantly higher MeJA and JA contents than WT, whereas those of *SlWRKY80* knockout lines were significantly lower (Fig. 7A), indicating that the *SlWRKY80*-overexpressing lines can show significantly increased endogenous MeJA and JA contents in tomato in saline–alkali stress, while the knockout lines exhibited contrary results. Under saline–alkali stress, exogenous application of 22.5 μmol/l MeJA to the 80CR-3 knockout lines significantly increased the endogenous MeJA and JA contents of tomato seedlings after 24 h (Fig. 7B), and the relative expression levels of JA synthesis-related genes such as *SlLoxD* and *SlAOC* also significantly increased (Supplementary Data Fig. S11), indicating that exogenous MeJA can significantly increase the endogenous MeJA and JA contents of tomato seedlings. In the results of 80CR-3 and 80CR-3 + MeJA under saline–alkali stress (Fig. 4), GUS staining results (Fig. 2C), Spd content (Fig. 5A), and Na^+^/K^+^ transport (Fig. 6A-C), both saline–alkali stress and exogenous MeJA treatment showed that they activated the promoter of *SlWRKY80* and led to its expression. *SlWRKY80* overexpression also promoted the expression of *SlSPDS2* (Supplementary Data Fig. S9) and *SlNHX4* (Supplementary Data Fig. S10). We also affirmed that *SlWRKY80* can directly regulate the promoters of *SlSPDS2* and *SlNHX4*, which implies that saline–alkali stress and MeJA exogenous spray actively regulate saline–alkali stress by increasing the endogenous MeJA and JA levels.

Allele oxide synthase (AOS), allele oxycyclinase (AOC), and 12-oxo plant dienoic acid reductase (OPR) are the rate-limiting enzymes in the JA biosynthetic pathway, while coronatine insensive1 (COI1) and JAZ proteins are two important receptors in the JA biosynthetic pathway [59]. JAZ protein contains the jasmonate Zim domain. As an inhibitor, the JA signaling pathway can be connected to other signaling pathways through combinations with transcription factors or other coenzyme proteins [5]. There are 13 genes in the tomato SlJAZ family, so we verified the interaction of SlWRKY80 with SlJAZ1, SlJAZ2, and SlJAZ5 at protein level through the Y2H assay (Fig. 7C). We verified it by LCI (Fig. 7D), BiFC (Fig. 7E), and pull-down assays (Fig. 7F) *in vivo* and *in vitro*. We found that only SlJAZ1 could get positive results under verification by these molecular means. Therefore, we conclude that there is a protein-level interaction between SlWRKY80 and SlJAZ1.

An SCF^COI1^ complex is formed when COI1 combines with JAZ protein, which results in JAZ protein degradation by the 26S proteasome, then releases transcription factors that interact with JAZ, thereby activating the expression of JA-responsive genes. Therefore, JA content is negatively correlated with the expression of SlJAZs [30, 50]. The same results were obtained in our study. When the synthesis of JA in tomato was inhibited, the expression of *SlJAZ1* was significantly upregulated, while the relative expression of *SlWRKY80*, *SlSPDS2*, and *SlNHX4* was opposite (Fig. 8C), indicating that the increase of endogenous JA content inhibited the expression of *SlJAZ1*. The interaction between SlJAZ1 as a transcriptional suppressor gene and SlWRKY80 will also be weakened, thus releasing more SlWRKY80 protein. This result was also proved by the dual luciferase test (Fig. 8B).

To sum up, saline–alkali stress or exogenous spraying with a certain concentration of MeJA could increase the contents of endogenous MeJA and JA in tomato. As a signal molecule, MeJA combined with the *SlWRKY80* promoter to promote the expression of *SlWRKY80*. On the other hand, the increase of endogenous JA content inhibited the expression of *SlJAZ1* and further released SlWRKY80 protein, interacting with SlJAZ1. At the same time, SlWRKY80 combined with the promoters of *SlSPDS2* and *SlNHX4* to promote Spd synthesis and Na^+^/K^+^ homeostasis, thus actively regulating saline–alkali stress.

## Materials and methods

### Plant materials

To verify the optimal concentration of exogenous MeJA under 300 mM saline–alkali stress, WT ‘Ailsa Craig’ (AC) tomatoes were used, and AC was also used as the background material to construct *SlWRKY80-*overexpressing lines (80OE-1 and 80OE-3) and *SlWRKY80* knockout lines (80CR-3 and 80CR-4). In addition, the JA synthesis mutant *spr8* (Solyc03g122340) used in this experiment was provided by Professor Li Chuanyou (Institute of Genetics and Developmental Biology, Chinese Academy of Sciences), and the background material for the knockout lines was CM WT tomato.

### Obtaining and identification of *SlWRKY80*-overexpressing and knockout lines

Overexpression vectors were constructed from tomato AC and pHellsgate2 (CaMV35S promoter driver) overexpressing *SlWRKY80* by cloning the CDS with SmaI and KpnI (an HA label was added to the pHellsgate2 vector, which was modified by Zhan Xiangqiang, School of Horticulture, Northwest A&F University). *SlWRKY80* target information was predicted through CRISPR RGEN Tools (http://www.rgenome.net/casdesigner/result?hash=49d4ef08e39c96a14781bc8f463be7f6) and constructing CRISPR/Cas9:*SlWRKY80*. Sequencing was performed by Sangon Biotech (Shanghai, China). *SlWRKY80* from different genotypes was compared with DNAMAN v.6 (Lynnon Biosoft, CA, USA). The primers used are listed in Supplementary Data Table S1.

The above-mentioned recombinant vectors were utilized to obtain transgenic materials via *Agrobacterium*-mediated infection [65]. Identification was performed using specific primers (Supplementary Data Table S1), and positive materials were retained for seed collection and continued propagation. The *T*_2_ generations of homozygous overexpressing (80OE-1, 80OE-3) and knockout lines (80CR-3, 80CR-4) were obtained by self-crossing to obtained transgenic lines.

### Plant growth conditions

This experiment was conducted in a growth chamber (model GXZ-5000E, China Ningbo Southeast Instrument Co., Ltd) under controlled conditions, with a daily light period of 12 h at 200 μmol m^−2^ s^−1^, temperature 25°C (day) and 20°C (night), humidity set to 60%. At the age of 15 days, we selected seedlings with consistent growth and planted them in a nutrient bowl and treated them at the age of 35 days (approximately five true leaves).

### Treatments

In the saline–alkali tolerance assay, a 300 mM composite saline–alkali solution (NaCl:Na_2_SO_4_:NaHCO_3_:Na_2_CO_3_ = 1:9:9:1, molar content ratio, pH = 8.6 ± 0.1) was used based on extensive saline–alkali stress resistance testing conducted by our research team [33, 56, 67, 68].

We verified the effect of exogenous MeJA at different concentrations on tomato stress resistance under saline–alkali stress. In the present study we sprayed MeJA at 0, 12.5, 22.5, 45, and 90 μmol/l in WT as a preliminary experiment and based on the report of Min *et al*. [42]. AC tomato seedlings at 35 days of age were irrigated with 100 mL of 300 mM saline–alkali solution, while external MeJA was applied by spraying. The concentrations of external MeJA were 0 (WT), 12.5, 22.5, 45, and 90 μmol/l. Each sprayed plant was 5 cm away from the tomato once, front, back, left, right. On average, ~5 ml of exogenous MeJA was sprayed per plant.

To investigate the effects of saline–alkali stress and exogenous MeJA on *SlWRKY80* expression and the promoter of *SlWRKY80*, we set up three groups of treatments, namely WT (control), S, and S + M. The WT (control) plants were irrigated with distilled water (100 ml) and the S (saline–alkali treatment) plants were irrigated with 300 mM saline–alkali (100 ml), while the S + M (saline–alkali and MeJA co-treatment) plants were irrigated with 300 mM saline–alkali solution (100 ml) and sprayed with 22.5 μmol/l of exogenous MeJA (CAS No. 39924-52-2, Sigma–Aldrich, USA). AC seedlings were treated at 0, 3, 6, 12, and 24 h, and the third leaf from the top of the tomato plant to measure the relative expression level of *SlWRKY80*. The pCAMBIA1391-pro-*SlWRKY80* material was subjected to GUS staining at 24 h of treatment.

A saline–alkali stress study was conducted to verify the function of *SlWRKY80*. We subjected AC (WT), *SlWRKY80*-overexpressing, and knockout lines from the same growth period to 300 mM saline–alkali treatment, and observed the phenotype 8 days later. We verified whether exogenous application of MeJA reduced the sensitivity of the 80CR-3 lines to saline–alkali stress. We selected AC and 80CR-3 tomato seedlings (35 days age) and grouped them into three treatments, namely WT, 80CR-3, and 80CR-3 + MeJA. Using AC tomato as WT, we poured 100 ml of saline–alkali solution on all three groups, and sprayed water (80CR-3) and an equal volume of 22.5 μmol/l MeJA (80CR-3 + MeJA) on 80CR-3. This experiment lasted for 8 days, during which the differences between each group were observed on a daily basis.

Further verification of the relationship between *SlWRKY80*, *SlSPDS2*, *SlNHX4*, and *SlJAZ1* was performed. We were fortunate enough to obtain a mutant *spr8* (*SlLoxD* gene mutant tomato material) synthesized from JA (using CM WT tomato as the background material). Based on this, we used CM WT tomato as the material, treated it with 300 mM saline–alkali, and applied 22.5 μmol/l MeJA and 22.5 μmol/L fluridone (JA synthesis inhibitor) (CAS No. CF5275, Beijing, China), externally, while spraying the same volume of ddH_2_O on *spr8*.

### RNA extraction and real-time quantitative PCR

Using 35-day-old WT, 80OE-1, 80OE-3, 80CR-3, and 80CR-4 tomato seedlings, we selected the third leaf from the top of the tomato plant, extracted RNA from the stem tissue and the entire root system, and measured the relative expression level of *SlWRKY80*. RNA extraction and first-strand cDNA synthesis and RT–qPCR were carried out following the protocol established by Xu *et al*. [65] and Livak *et al*. [37]. Related primers are provided in Supplementary Data Table S2.

In the experiments using saline–alkali treatment (S) and saline–alkali and MeJA co-treatment (S + M), we collected leaf tissue samples at 0, 6, and 12 h of treatment, and extracted RNA to measure *SlWRKY80* expression. The sampling position was the third blade from the top.

### Determination of morphological and physiological indicators

The phenotypes of the WT and *SlWRKY80* transgenic lines treated with a 300 mM saline–alkali solution were observed, and various physiological parameters were determined on the eighth day. A ruler, vernier caliper, and root scanner (Perfection V700N, Epson Co., Ltd, China) were used to measure aboveground morphological indicators [68]. The formula for calculating the sound seedling index was: (stem diameter/plant height + root dry weight/aboveground dry weight) × whole plant dry weight.

The physiological indicators included the activities of SOD, POD, and CAT, proline content, malondialdehyde content, and so on. A reagent kit was used to measure the physiological indicators (Nanjing Jiancheng Biotechnology Research, Nanjing, China).

### Histochemical GUS activity assay

To clarify the response of the promoter of *SlWRKY80* to MeJA, JA-Ile and saline–alkali, the pCAMBIA1391-pro-*SlWRKY80* vector was constructed using HindIII and SalI as restriction endonuclease sites and transferred into AC tomato plants through genetic transformation, and pCAMBIA1391-pro-*SlWRKY80*-positive material was obtained. The relevant primers are shown in Supplementary Data Table S1.

Untreated pCAMBIA1391-pro-*SlWRKY80*-positive material was used as a control in this experiment. We performed S, M, S + M processing separately. After 24 h of treatment, GUS staining was performed referring to Liang *et al*. [29] (The decolorization process after GUS staining ends when the AC leaves are decolorized until colorless).

### Subcellular localization of *SlWRKY80*

The CDS of *SlWRKY80*, excluding the stop codon, was cloned into the pBI121-GFP vector at the SacI and BamHI restriction sites, resulting in the generation of the pBI121-*SlWRKY80*-GFP construct. Additionally, the pBI121-GFP construct was produced using the same SacI and BamHI restriction sites. The primers used are mentioned in Supplementary Data Table S1. For subcellular localization, the pCMV-C-mCherry vector (Beyotime, Shanghai, China) was employed. The plasmids were introduced into *Agrobacterium tumefaciens* GV3101 utilizing the freeze–thaw method. Transient transformation of *Nicotiana benthamiana* leaf epidermal cells was subsequently conducted. Detailed operation methods can be found in Xu *et al*. [66].

### Determination of free spermidine content

On the eighth day of treatment, the second leaf from the top was sampled, with three biological replicates from each group. The determination of free Spd content was carried out using the method of Wang *et al*. [56].

### Determination of Na^+^ and K^+^ contents

On the eighth day of treatment, samples were collected from roots, stems, and leaves. The entire root system of the tomato was selected, and the stem was selected at a distance of 1–3 cm from the root system. The second true leaf from the top was selected, and each group of treatments underwent three biological replicates. Na^+^ and K^+^ contents were determined using Wang *et al*.’s method [58].

### Determination of endogenous hormone content

JA and MeJA contents in tomato leaves were determined by taking samples from the second true leaf from the top 0 and 24 h post-treatment. The determination of endogenous hormone content was carried out using the method of Xu *et al*. [66].

### Yeast one-hybrid assay

*SlWRKY80* cDNA was sequenced and cloned into pGADT7 vector for construction of prey-*SlWRKY80*. Three distinct fragments of the *SlSPDS2* promoter, along with the *SlNHX4* promoter, were individually inserted into the pAbAi vector to construct pBait-reporter vectors. Upon completing vector construction, the Y1H procedure was conducted in accordance with the methodology outlined by Liang *et al*. [29]. Supplementary Data Table S1 lists the related primers.

### Chromatin immunoprecipitation qPCR assay

Total protein extracts from WT and 80OE-13 leaves on pre- and post-saline- alkali plants were subjected separately to ChIP assays using a ChIP kit (Beyotime). HA antibodies were used to immunoprecipitate DNA–protein complexes (Sigma). After the precipitated complexes were recovered, ChIP–qPCR assays using the primers provided in Supplementary Data Table S2 were performed.

### Electrophoretic mobility shift assay

The CDS of *SlWRKY80* was cloned into the pGEX4T-1 expression vector using the EcoRI and SalI sites to produce a glutathione *S*-transferase fusion protein. Related primers can be found in Supplementary Data Table S1. The fusion protein was then transformed into *Escherichia coli* line BL21, and induction was carried out using constant shaking at 200 revolutions/min and a temperature of 28°C for 8 h with an IPTG concentration of 0.5 mM. A method based on glutathione Sepharose beads (635608, Takara) was then used to purify the fusion protein. Invitrogen synthesized the biotin-labeled *SlSPDS2* and *SlNHX4* promoter oligonucleotide probes, which are listed in Supplementary Data Table. EMSAs were performed utilizing a LightShift Chemiluminescent EMSA Kit (Beyotime). The mutation and cold probes employed for EMSA can be found in Supplementary Data Table S3.

### Yeast two-hybrid assay

To assess the self-activation of *SlWRKY80*, we divided it into two fragments consisting of a conserved domain, which were subsequently cloned separately into the yeast pGBKT7 vector, with BD-*SlWRKY80*_202-819bp_ and BD-*SlWRKY80*_1-819bp_ cleavage sites. Supplementary Data Table S1 lists the primer sequences. BD-*SlWRKY80*_202-819bp_, BD-*SlWRKY80*_1-819bp_, and pGADT7 were transformed into Y2H. After transformation, 50 μl of the transformed cells was placed onto solid-state two-deficient DDO (SD/−Trp/−Leu) and four-deficient QDO (SD/−Trp/−Leu/−His/−Ade) media. The media were incubated for 3–5 days at 28°C, and yeast growth was recorded. This showed that BD-*SlWRKY80*_202-819bp_ was a non-self-activating fragment containing the structural domain of *SlWRKY80*.

The CDSs of 13 genes from the SlJAZ family in tomato were cloned into yeast pGADT7 vector with NdeI and XhoI cleavage sites, and then co-transformed with BD-*SlWRKY80*_202-819bp_ into Y2H. After transformation, 50 μl of the transformed cells was placed onto solid-state media of two-deficient DDO and four-deficient QDO. The media were placed in an incubator at 28°C for 3–5 days.

### Bimolecular fluorescence complementation assay

The full-length CDSs of *SlWRKY80* and *SlJAZ1* (excluding the termination codon) were constructed in the pSPYNE and pSPYCE vectors, respectively. Supplementary Data Table S1 provides the primers. Subsequently, the constructed vector was sequenced and validated, and the plasmid was transferred into *Agrobacterium* GV3101. The empty plasmids pSPYNE and pSPYCE, along with the recombinant plasmid-containing *Agrobacterium*, were diluted with MES (OD_600_ = 1.0). Then, *Agrobacterium* containing pSPYNE-X (Vec or *SlWRKY80*) and pSPYCE (Vec or *SlJAZ1*) was mixed in a 1:1 volume ratio and left to stand in a dark environment at 28°C for 2 h before injection into tobacco leaves. After 48 h, the infected tissue was examined using a confocal microscope (LAS X, Leica, Mannheim, Germany). The images were then subjected to postprocessing using Leica LAS X software (v.3.7.2). The scale used in this experiment was 20 μm.

### Luciferase complementary imaging experiment

The CDS of *SlWRKY80* was constructed into the N-terminus of JW771 (N-Luc), and *SlJAZ1* was constructed into the C-terminus of JW772 (C-Luc). Supplementary Data Table S1 provides the primers. Subsequently, the recombinant plasmid with accurate sequencing was transferred into GV3101. The empty plasmids JW771 and JW772, as well as the recombinant plasmid-containing *Agrobacterium*, were diluted with infection buffer (OD_600_ = 1.0). Then, *Agrobacterium* containing JW771-X (Vec or *SlWRKY80*) and JW772 (Vec or *SlJAZ1*) was mixed in a 1:1 volume ratio and left to stand in a dark environment at 28°C for 2 h before injection into tobacco leaves. After 48 h, 0.5 mM d-luciferin was evenly applied to the back of tobacco leaves, and photographs were taken using a plant living molecule labeling imaging system (CCD). The scale was 20 μm.

### Pull-down assay

Induction and purification of the GST­-SlWRKY80 fusion protein is described above in the section ‘Electrophoretic mobility shift assay'. Cloning of the CDS of *SlJAZ1* into pMAL-C2x was performed using EcoRI and HindIII. The expression vector was generated using relevant primers (Supplementary Data Table S1) to produce the MBP-*SlJAZ1* fusion protein. Next, the *E. coli* BL21 line was transformed with the fusion protein and grown by shaking the culture at 200 revolutions/min and 28°C for 8 h, with induction using IPTG at a concentration of 0.5 mM. Subsequent removal of the medium was performed by centrifugation (temperature 4°C, speed 4000 rpm, duration 10 min), and the supernatant was discarded. The remaining substance was resuspended in 1 × PBS (pH = 8.0) and subsequently subjected to ultrasound treatment on ice for 15 min. The solution was then centrifuged again (temperature 4°C, speed 4000 rpm, duration 10 min), and the supernatant was collected. A maltose binding protein label protein purification kit was used to further purify the obtained MBP-*SlJAZ1* fusion protein (Abbkine Biotechnology Co., Ltd, Wuhan, China).

The GST pull-down analysis method used was as described by Zhang *et al*. [72].

### Dual-luciferase analysis

The full-length CDS of *SlWRKY80*, excluding the termination codon, was cloned into the pGreenII-002962-SK vector using SacI and KpnI cleavage sites. The *SlSPDS2* promoter has three promoter segments while the *SlNHX4* promoter has one promoter segment containing the W-box. They were constructed in the pGreen-II-0800 vector, with KpnI and NcoI enzyme cleavage sites. The primers used are listed in Supplementary Data Table S1. The dual luciferase test operation was based on the method of Liang *et al*. [29]. The tobacco plants were then incubated for 72 h before fluorescence detection was performed using the Dual Lucifera Reporter Assay System (E1910, Promega, USA).

### Statistical analysis

The LSD test, based on DPS7.5, was used to detect significant differences between three replicates of each experiment.

## Supplementary Material

Web_Material_uhae028

## Data Availability

The authors affirm that all the information required to substantiate the findings of the study is provided in both the paper and the supplementary materials, or can be acquired by contacting the corresponding author.

## Accession numbers

A database of online sequence data was used to obtain sequence data (https://solgenomics.net/search/locus?tdsourcetag=spcqq_aiomsg).

The gene accession numbers used in this study can be found below: Solyc03g095770.2 (*SlWRKY80*), Solyc01g098190.3 (*SINHX4*), (*SlSPDS2*), Solyc07g042170.3 (*SlJAZ1*), Solyc12g009220.2 (*SlJAZ2*), Solyc03g122190.3 (*SlJAZ3*), Solyc12g049400.2 (*SlJAZ4*), Solyc03g118540.3 (*SlJAZ5*), Solyc01g005440.3 (*SlJAZ6*), Solyc11g011030.2 (*SlJAZ7*), Solyc06g068930.2 (*SlJAZ8*), Solyc08g036640.3 (*SlJAZ9*), Solyc08g036620.3 (*SlJAZ10*), Solyc08g036660.3 (*SlJAZ11*), Solyc01g009740.2 (*SlJAZ12*), Solyc01g103600.3 (*SlJAZ13*), Solyc03g122340.2 (*SlLoxD*), Solyc02g085730.2 (*SlAOC*).
